# Supplementary material for: Concordant and Discordant Cerebrospinal Fluid and Plasma Cytokine and Chemokine Responses in Mild Cognitive Impairment and Early-Stage Alzheimer’s Disease
Source: Biomedicines. 2023 Aug 27;11(9):2394. doi: 10.3390/biomedicines11092394 (PMC10525668; doi:10.3390/biomedicines11092394)
Supplement: Supplementary file 1 [file biomedicines-11-02394-s001.zip › biomedicines-2545207-supplementary.pdf]

Supplementary Table S1: Serum Cytokine/Chemokine Multiplex ELISA: ANOVA Results

Cytokine/Chemokine	F-Ratio	P-Value	MCI Effect	AD Effect
basic-FGF	2.94	*0.064*		**Dec**
Eotaxin	6.77	**0.003**	**Inc**	**Inc**
G-CSF	1.038	N.S.		
GM-CSF	19.69	**<0.0001**	**Dec**	**Dec**
IFN-γ	0.115	N.S.		
IL-10	0.646	N.S.		
IL-12p70	0.022	N.S.		
IL-13	8.32	**0.0009**	**Inc (Inc)**	**Inc**
IL-15	24.93	**<0.0001**	**Dec**	**Dec**
IL-17a	19.48	**<0.0001**	**Inc**	**Inc**
IL-1β	3.211	*0.051*	*Inc*	*Inc*
IL-1rα	0.368	N.S.		
IL-2	17.19	**<0.0001**	**Dec**	**Dec**
IL-4	65.27	**<0.0001**	**Inc**	**Inc**
IL-5	110.18	**<0.0001**	**Inc**	**Inc**
IL-6	2.107	N.S.		
IL-7	0.388	N.S.		
IL-8	1.046	N.S.		
IL-9	1.235	N.S.		
IP-10	2.605	*0.087*	*Dec*	*Dec*
MCP-1	3.070	**0***.058*	*Dec*	**Dec**
MIP-1α	3.834	**0.030**	*Inc*	**Inc**
MIP-1β	0.860	N.S.		
PDGF-bb	33.70	**<0.0001**	**Inc**	**Inc**
RANTES	10.80	**0.0002**	**Inc**	**Inc**
TNF-α	8.639	**0.0008**	**Inc**	**Inc**
VEGF	0.494	N.S.		

Multiplex Bead-based ELISA results from assay of serum from 21 controls, 8 subjects with MCI, and 10 subjects with AD. See Table 1 for Factor full names and functions. Data were analyzed by one-way repeated measures ANOVA with the post hoc Tukey-Kramer

Multiple Comparison Test of significance. F-Ratios and significant P-values (P<0.05) are in bold font, whereas statistical trends (0.05<P<0.10) are italicized. N.S. = not statistically significant. Significant post hoc differences and directional changes (Inc= increased; Dec= decreased) from control are indicated. Significantly higher levels of IL-13 were observed in MCI versus AD. Otherwise, the MCI and AD results were comparable with similar directional shifts in cytokine/chemokine expression relative to control.

Supplementary Table S2: CSF Cytokine/Chemokine Multiplex ELISA: ANOVA Results

Cytokine/Chemokine	F-Ratio	P-Value	MCI Effect	AD Effect
basic-FGF	8.44	**0.0093**	**Dec**	**Dec**
Eotaxin	0.287	N.S.		
G-CSF	0.563	N.S.		
GM-CSF	56.88	**<0.0001**	**Dec**	**Dec**
IFN-γ	2.242	N.S.		
IL-10	0.537	N.S.		
IL-12p70	0.734	N.S.		
IL-13	3.323	**0.046**		**Inc**
IL-15	0.984	N.S.		
IL-17a	9.431	**0.0005**	**Dec**	**Dec**
IL-1β	0.068	N.S.		
IL-1rα	0.164	N.S.		
IL-2	0.076	N.S.		
IL-4	1.271	N.S.		
IL-5	4.333	**0.02**	**Inc**	**Inc**
IL-6	1.249	N.S.		
IL-7	3.148	*0.054*	**Inc**	
IL-8	0.420	N.S.		
IL-9	1.728	N.S.		
IP-10	1.043	N.S.		
MCP-1	1.336	N.S.		
MIP-1α	0.749	N.S.		
MIP-1β	1.069	N.S.		
PDGF-bb	10.15	**0.0003**	**Dec**	**Dec**
RANTES	0.422	N.S.		
TNF-α	1.318	N.S.		
VEGF	37.06	**<0.0001**	**Dec**	**Dec**

Multiplex Bead-based ELISA results from assay of CSF from 21 controls, 8 subjects with MCI, and 10 subjects with AD. See Table 1 for Factor full names and functions. Data were analyzed by one-way repeated measures ANOVA with the post hoc Tukey-Kramer

Multiple Comparison Test of significance. F-Ratios and significant P-values (P<0.05) are in bold font, whereas statistical trends (0.05<P<0.10) are italicized. N.S. = not statistically significant. Significant post hoc differences and directional changes (Inc= increased; Dec= decreased) from control are indicated. Significantly higher levels of IL-13 were observed in MCI versus AD. Otherwise, the MCI and AD results were comparable with similar directional shifts in cytokine/chemokine expression relative to control.
